# Triple-Negative Apocrine Breast Carcinoma Has Better Prognosis despite Poor Response to Neoadjuvant Chemotherapy

**DOI:** 10.3390/jcm11061607

**Published:** 2022-03-14

**Authors:** Taobo Hu, Yiqiang Liu, Jinbo Wu, Xuejiao Lina Hu, Guiyang Zhao, Baosheng Liang, Shu Wang, Mengping Long

**Affiliations:** 1 Department of Breast Surgery, Peking University People’s Hospital, Beijing 100032, China; thuac@connect.ust.hk (T.H.); sweet7766@163.com (J.W.); 2 Department of Pathology, Peking University Cancer Hospital, Beijing 100036, China; victor.liu76@163.com; 3 Department of Pathology, Alaska Native Medical Center, Anchorage, AK 99501, USA; nini114@gmail.com; 4 Department of Oncology, Beijing Changping Hospital, Beijing 100192, China; 18611002258@163.com; 5 Department of Biostatistics, School of Public Health, Peking University, Beijing 100080, China; liangbs@hsc.pku.edu.cn

**Keywords:** invasive apocrine carcinoma, triple-negative breast cancer, androgen receptor, neoadjuvant therapy

## Abstract

Apocrine carcinoma is a rare subtype of invasive ductal breast cancer that shows apocrine differentiation and largely triple-negative immunohistology. Triple-negative breast cancers are known to have more aggressive clinical courses. However, unlike most other subtypes, it is reported that triple-negative apocrine carcinoma (TNAC) has a better prognosis. Due to the scarcity of reported studies, our knowledge regarding its clinical behavior, prognosis and response to therapy is very limited. In this study, we retrospectively retrieved 41 triple-negative apocrine carcinoma cases from our breast cancer database, with an average follow-up of 32.8 months. It was found that TNAC had a poorer response to neoadjuvant therapy but a better prognosis than other nonapocrine types of triple-negative breast cancer. Meanwhile, TNAC has a low proliferative nature, as indicated by its low Ki-67 index. An updated analysis of the Surveillance, Epidemiology, and End Results database showed that chemotherapy did not improve breast-cancer-specific survival in TNAC patients. Our results suggest that TNAC is a special subtype of triple-negative breast cancer with a better short-term prognosis despite poor response to neoadjuvant chemotherapy.

## 1. Introduction

Apocrine carcinoma is a rare histologic subtype of breast cancer, accounting for approximately 1% of all breast cancers, and is diagnosed by the apocrine differentiation of the cancer cells [1]. Apocrine carcinomas are often estrogen receptor (ER)- and progesterone receptor (PR)-negative, with 30% of them having human epidermal growth factor 2 (HER2) amplification [2]. Thus, the majority of apocrine carcinomas are triple-negative, which means that they lack the expression of ER, PR and HER2 and can be named triple-negative apocrine carcinoma (TNAC).

Although TNAC does not express ER and PR, it strongly expresses the androgen receptor (AR) and is activated in AR pathway [1,2]. The exact effect of AR expression on TNAC has not been elucidated, together with the question of whether anti-AR therapy can be applied in TNAC patients, whereas in triple-negative breast cancers (TNBC) of nonapocrine subtype, the expression of AR and its consequent effects are well documented. AR is expressed in 10–63% of TNBC [3,4], and its expression is correlated with good prognosis in early-stage breast cancer in terms of both disease-free survival (DFS) and overall survival (OS) [5,6]. Molecularly, the high expression of AR is a characteristic of the luminal androgen receptor (LAR) subtype, which is one of the four molecular subtypes of TNBC classified using transcriptomic data [7,8,9,10]. LAR, which accounts for 15–20% of all TNBC cases, has been reported to have a low proliferative rate, and distant metastases often develop after three years [11]. Approximately 59% of LAR cases were found to show histologic apocrine differentiation in more than 10% of all the cancer cells [12]. TNAC shares certain common features with the LAR subtype, including a low proliferative nature and a better prognosis than other TNBC cases [13,14]. LAR cell lines are sensitive to AR inhibitors, including bicalutamide and enzalutamide [9,15]. However, the response of TNAC to anti-AR therapy and the exact correlation between TNAC and the LAR subgroup is still largely unknown due to the limited reported TNAC cases.

Despite the apparent discrepancy between TNAC and TNBC of the nonapocrine subtype in morphology and molecular features, they are currently treated in the same way. However, accumulating evidence suggests that TNAC patients have limited benefit from conventional chemotherapy. A study comparing TNAC patients who did not receive adjuvant chemotherapy with matched TNBC patients who received adjuvant chemotherapy showed that the two groups had similar prognoses, which indicates potential for de-escalation in the management of TNAC [16]. Nagao et al. reported that of five invasive apocrine carcinoma patients who received neoadjuvant chemotherapy, none achieved a pathological complete response (pCR) [17]. However, for TNAC patients, the clinical evidence for the response to neoadjuvant chemotherapy is lacking.

In this study, we compared the clinicopathological characteristics and survival of 41 TNAC cases with paired TNBC cases, 21 of which had received neoadjuvant therapy. The response was evaluated with the Miller–Payne grading system and Residual Cancer Burden (RCB) index.

## 2. Methods

### 2.1. Study Population

The study was approved by the Peking University Cancer Hospital ethics committee (reference number 2020KT113). The pathology database of the Peking University Cancer Hospital was queried for breast apocrine carcinomas diagnosed between 2008 and 2021. A total of 41 cases that met the diagnostic criteria—including at least 95% of the tumor showing apocrine differentiation, the N:C ratio of tumor cells being 1:2 or more with abundant eosinophilic cytoplasm, prominent nucleoli, and sharply defined cell borders—were included and independently verified by two pathologists. Patients with only apocrine carcinoma in situ were excluded. These 41 patients also had tumor surgical resection data to achieve accuracy for pathological staging. The ER, PR and HER2 statuses were evaluated by immunohistochemical staining and fluorescent in situ hybridization (FISH). Each of the TNAC cases was paired with one nonapocrine triple-negative breast cancer (TNBC-NA) case by age at diagnosis, year of diagnosis and receipt of neoadjuvant therapy to exclude the effect of bias on prognosis. All of the paired TNBC-NA cases were invasive breast cancer of no special type. None of the included cases had distant metastasis at the initial diagnosis. Of the 41 cases of TNAC, 21 had received neoadjuvant therapy and were included in the neoadjuvant comparison group. The other 20 cases were included in the non-neoadjuvant group, and all of them had received adjuvant chemotherapy. The survival was analyzed by Kaplan–Meier estimation, and the responses of 21 TNAC cases that received therapy were evaluated with the Miller–Payne grading system and RCB index.

### 2.2. Immunohistochemical Staining

Immunohistochemistry was performed on the formalin-fixed, paraffin-embedded tissue following the manufacturer’s instructions of each specific antibody. Antibody used: ER (SP1, Roche, 1 μg/mL), PR (1E2, Roche, 1 μg/mL), HER2 (4B5, Ventanne, 6 μg/mL and 18299-1-AP, Proteintech, 1:1000), Ki-67 (M1B1, Zhongshanjinqiao, working concentration), EGFR (EP22, Jinbiaoyatu, working concentration), AR (EP120, Zhongshanjinqiao, working concentration), GCDFP-15 (EP95, Zhongshanjinqiao, working concentration). The immunohistochemical stains were evaluated by two pathologists with consensus (M.L. and Y.L.). The Ki-67 score is defined as the percentage of positively nuclear-stained cells divided by the total number of malignant cells scored. When the staining is homogenous across the sample, the global Ki-67 score is used. For heterogeneous staining, the consensus guideline recommends that the overall score together with the score in hotspot regions should be reported [18]. For practical data analysis, the Ki-67 score of hotspot regions is reported when heterogeneity is noticed in this study [19].

### 2.3. SEER Analysis

TNAC cases from the SEER database were obtained using the SEER*Stat software, version 8.3.9.2. A total of 442 TNAC patients were identified from 2010 to 2018 according to the following selection criteria: invasive apocrine carcinoma aged over 18 years old, negative ER and PR statuses, a negative HER2 status, and detailed information about survival being available. Propensity score matching (PSM) was used to achieve 1:1 matching between the chemotherapy and non-chemotherapy groups, to reduce the compound effects caused by baseline information bias. The R package “MatchIt” was employed for PSM [20].

## 3. Results

### 3.1. Patients with TNAC Have Better Short-Term Prognosis Than Those with TNBC-NA Despite a Poorer Response to Neoadjuvant Chemotherapy

**Figure 1:**
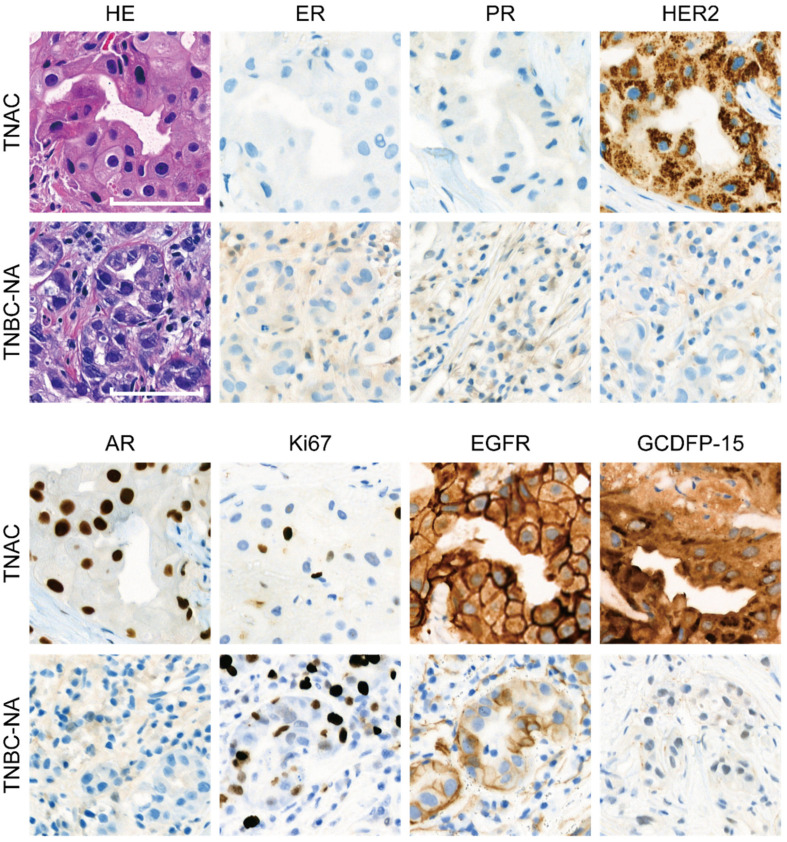
Representative HE and IHC staining images from TNAC and TNBC-NA patients. Scale bar indicates 50 μm.

The immunohistochemical staining of protein markers including ER, PR, HER2, Ki-67, AR, EGFR and GCDFP-15, together with the hematoxylin and eosin staining in the breast cancer tissue of the TNAC and TNBC-NA groups, is illustrated in Figure 1. Interestingly, in 11 cases in the TNAC group (11/41, 27%), the original HER2 immunostaining using an anti-HER2 antibody (clone 4B5, Ventana, 6 μg/mL) and BenchMark automatic IHC system (Ventana Medical System, Tucson, AZ, USA) showed an intense cytoplasmic granular signal, which was reported as equivocal 2+ or 1+ (Figure 1). Meanwhile, further FISH proved all the HER2 to be nonamplified. Moreover, when another clone of the HER2 antibody (18299-1-AP, Proteintech, 1:1000) was applied for immunohistochemical staining, the cytoplasmic signal disappeared, which indicated that the cytoplasmic signal could be nonspecific. The clinicopathologic characteristics of the 21 TNAC cases that did not receive neoadjuvant therapy and their paired TNBC-NA cases are summarized in Table 1. Those who received the neoadjuvant therapy group are displayed in Table 2. There is no significant difference in terms of T stage, N stage and AJCC stage between TNAC and TNBC groups indicating that the paring is also stage-balanced and stage would not be a bias factor in later survival analysis. The median age at diagnosis for the TNAC group was 57 years (range: 36–77 years). Among them, 15 patients (15/41, 36.5%) had received lumpectomy, and the other 26 patients (26/41, 63.5%) received a mastectomy. The Ki-67-positive percentage and the percentage of tumor-infiltrating lymphocytes (TILs) were evaluated using the biopsy sample before any treatment. The mean Ki-67-positive percentage of TNAC was significantly lower than the matched TNBC-NA in both the neoadjuvant group and the non-neoadjuvant group (*p* < 0.05) (Table 1 and Table 2). The histologic grade of the TNAC group was less advanced than that of the TNBC-NA group in the non-neoadjuvant group but was similar in the neoadjuvant group. For the percentage of stromal TILs, TNAC has a tendency towards showing fewer TILs compared with TNBC-NA, although this was not statistically significant in either the non-neoadjuvant or neoadjuvant group. Other clinicopathological features, including the surgery type and radiation therapy, showed no significant difference between TNAC and TNBC-NA.

**Figure 2:**
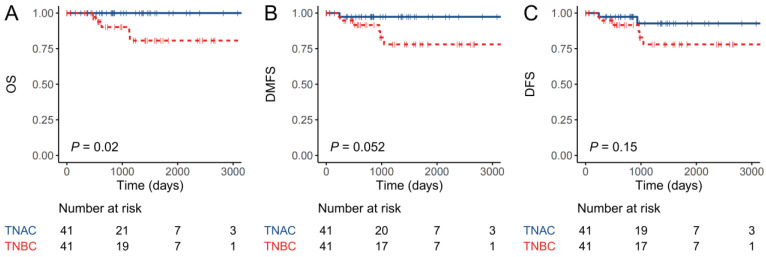
Survival plot for TNAC and TNBC-NA cohort. (**A**) Overall survival (OS), (**B**) distant metastasis-free survival (DMSF) and (**C**) disease-free survival (DFS) of TNAC and TNBC-NA groups were analyzed with Kaplan–Meier curve.

For survival analysis, the median time of follow-up was 32.8 months (range: 1.2–35.6 months). In the TNAC group, none of the 21 patients had died at the time of the last follow-up, while five patients in the TNBC-NA group (5/41, 12%) had died, all due to breast cancer. The OS of the TNAC patients was better than that of the TNBC-NA patients (*p* = 0.02) (Figure 2). According to the distant-metastasis-free survival (DMFS) analysis, only one patient in the TNAC group had experienced distant metastasis. Thus, the DMFS survival curves of the two groups showed the same trend as in OS, whereas the *p*-value was 0.052, which was marginally statistically significant, possibly due to the small sample size. The DFS which includes the local recurrence of the two groups showed no significant difference (Figure 2).

Twenty-one cases in the TNAC group had received neoadjuvant therapy, and the pathological response was evaluated using both the Miller–Payne (MP) grading system [21] and the RCB index [22]. The MP grade evaluates cancer response only in the primary tumor while the RCB index takes also into account the axillary lymph node status as the lymph node status after neoadjuvant therapy has been shown to have prognostic significance in breast cancer [23,24]. 

**Figure 3:**
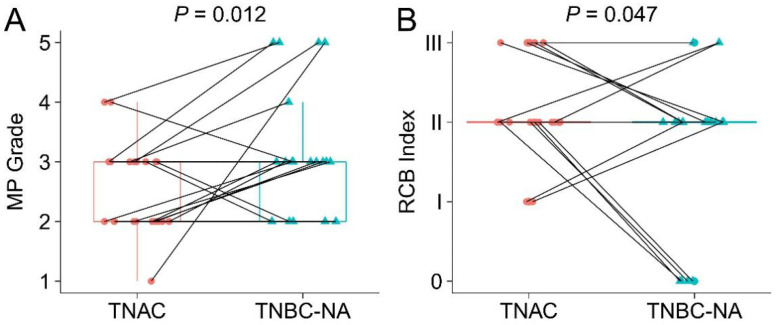
Paired Miller–Payne grading (**A**) and Residual Cancer Burden grading (**B**) of the study cohort. TNAC cases are plotted with red dots and TNBC-NA cases are plotted with green triangles. Each TNAC and TNBC-NA pair is connected with a black line. MP grade system is based on a 5-point scale system with point 5 indicating complete response and point 1 indicating no response. RCB index divided the residual disease into four categories: RCB-0 (pCR), RCB-I (minimal residual disease), RCB-II (moderate residual disease) and RCB-III (extensive residual disease). Statistical analysis was performed with a one-sided paired Wilcoxon test.

In the TNAC group, none of the 21 cases had achieved a pathological complete response (pCR), which corresponds to an RCB index of zero, while four patients (4/21, 19%) in the TNBC-NA group had achieved pCR. Both MP grade and RCB index comparison showed that the tumor response was poorer in TNAC than that in the matched TNBC-NA group (*p* = 0.012 and 0.047), as demonstrated in Figure 3. The detailed clinical information of the TNAC cases in the neoadjuvant group, including the neoadjuvant therapy regimen, clinical evaluation of response, tumor and lymph node stage, Ki-67-positive percentage, histologic grade, RCB index and TIL percentage, are listed in Table 3. The corresponding information of TNBC-NA cases is provided in Supplementary Table S1.

### 3.2. Chemotherapy did Not Improve Breast-Cancer-Specific Survival for TNAC Patients

**Figure 4:**
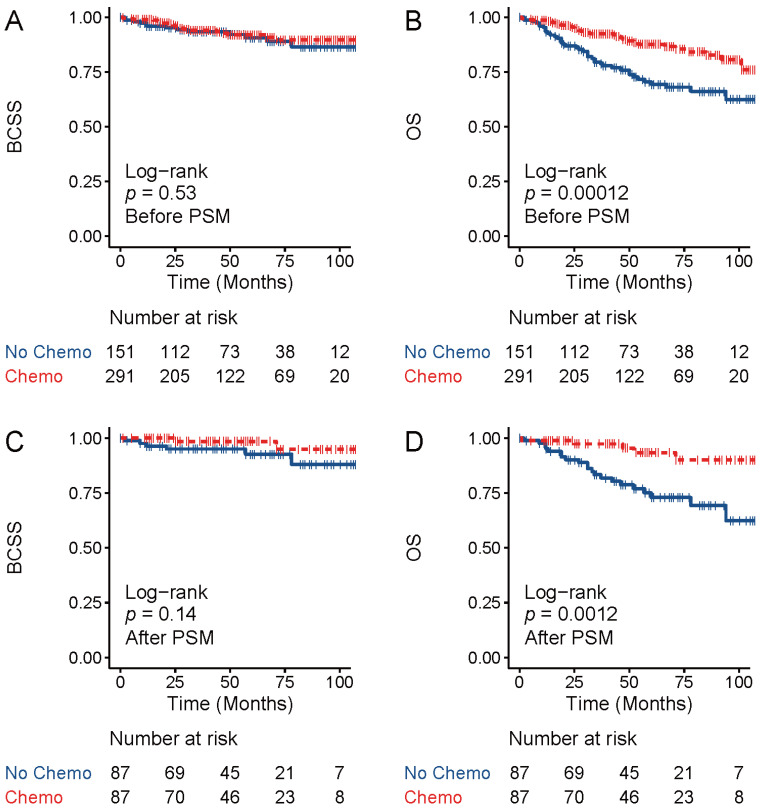
Survival plots of chemotherapy group and no-chemotherapy group of TNAC patients from SEER. Breast-cancer-specific survival (BCSS) and overall survival (OS) of TNAC patients before matching (**A**,**B**) and after matching (**C**,**D**) were analyzed with Kaplan–Meier curves. Numbers at risk are displayed below.

Although TNAC patients showed a poor response to neoadjuvant chemotherapy, whether patients with TNAC would benefit from chemotherapy remains unknown. Previous studies found that TNAC survival was better than survival among TNBC patients according to the SEER database [25,26,27]. Furthermore, Wu et al. reported that, in TNAC, patients with chemotherapy have better OS than those who do not undergo chemotherapy according to the SEER database [13]. However, their findings are limited by the absence of PSM and, thus, could be confounded by baseline bias. Moreover, the effect of chemotherapy on the breast-cancer-specific survival (BCSS) of TNAC patients has not been elucidated. We next investigated the effect of chemotherapy on the OS and BCSS of TNAC patients using the SEER database with and without PSM. A total of 442 patients with TNAC were enrolled in the study, which was divided into chemotherapy and no-chemotherapy groups according to whether they had received chemotherapy. There were 291 (65.8%) patients in the chemotherapy and 151 (34.2%) patients in the no-chemotherapy group. The demographic and clinicopathological information of the patients is displayed and compared in Table 4. The patients in the chemotherapy group presented with a younger age at diagnosis, more advanced stage status and higher histology grade than those in the chemotherapy group. Additionally, patients in the chemotherapy group were more likely to receive surgery and radiation therapy. To eliminate bias in the baseline information between the two groups, 1:1 matching was performed using propensity score matching. After matching, 87 patients remained in both groups, and there was no difference in demographic or clinicopathological features between the two groups. The BCSS and OS of the two groups both before and after matching were plotted as a Kaplan–Meier survival curve (Figure 4). Before matching, patients in the chemotherapy group showed better survival than the no-chemotherapy group, while there was no difference in BCSS between the two groups. For the two groups after PSM, the results were the same as those before matching, indicating that chemotherapy did not improve BCSS for the TNAC patients.

## 4. Discussion

Apocrine carcinoma is a special subtype of breast cancer characterized by apocrine metaplasia, histologically, and the activation of the AR pathway, molecularly. The mutational rate of the PI3KCA gene in TNAC is 72% higher than that in other TNBC, with a 55% mutation rate in the LAR group [28,29]. Despite these differences, TNAC is treated in the same manner as other TNBC, although the benefit of the AR antagonist bicalutamide was investigated in advanced ER- and PR-negative patients [30]. In concordance with previous studies [31,32,33,34], our results demonstrate that apocrine carcinoma has better prognosis than invasive breast carcinoma of no special type. More significantly, our results showed that TNAC patients have a poor chemotherapy responsiveness and no benefit from chemotherapy in terms of BCSS. Previous studies showed that pCR can be used as a surrogate endpoint in TNBC patients receiving neoadjuvant chemotherapy, meaning that patients who reached pCR have better survival than those who did not reach pCR [35,36,37,38,39,40]. Among the four molecular subtypes of TNBC, the LAR group has the lowest pCR rate [11]. In our study, none of the 21 TNAC cases had achieved pCR under various treatment regimens, suggesting TNAC as a specific subtype of AR-positive TNBC. Although, in this study, we matched TNAC with TNBC-NA cases to reduce selection bias, our study is still limited by its retrospective nature. However, conducting a random clinical trial with a rare disease is also challenging. Our study is also limited by its average follow-up time of 32.8 months, which could be relatively short for TNAC since previous studies showed that distant metastases in the LAR subtype of TNBC often develop after 3 years from the first diagnosis. A further study with a longer follow-up time is needed to compare the long-term prognosis of TNAC and TNBC. In further research with more TNAC cases, it would also be necessary to compare the outcomes of TNAC patients with different chemotherapy response to further clarify the role of chemotherapy on TNAC.

A previous study showed that, compared with luminal breast cancer, TNBC has higher sensitivity to anthracycline-based neoadjuvant chemotherapy but worse prognosis, which could be attributed to the higher relapse rate in non-pCR patients [41]. In this study, TNAC showed the same pattern as luminal breast cancer, which could be attributed to the expression of AR and other luminal genes in TNAC. AR-positive TNBC is known to have a more favorable prognosis than AR-negative TNBC, possibly due to the anti-proliferative effect of AR [42]. Meanwhile, anti-androgen-receptor therapeutics including bicalutamide, enzalutamide and abiraterone have shown a clinical benefit ratio ranging from 19% to 35% in AR-positive TNBC [43,44,45]. Thus, for the management of TNAC, the benefits of conventional chemotherapy and the possibility of adding anti-androgen receptor therapy need to be evaluated in future studies.

Last but not least, previous studies identified that compared to nonapocrine breast carcinoma, apocrine breast carcinoma cells were rich in mitochondria and secretory globules in the cytoplasm [46]. Mitochondria-related proteins including PGC1α and p62 were found to be overexpressed in apocrine carcinoma [47]. The nonspecific IHC staining of HER2 in the Ventana system observed in our study could be contributed by the cytoplasmic enrichment of mitochondrial proteins. It reminds us to be more careful when interpreting HER2 IHC staining in apocrine carcinoma to avoid unnecessary HER2 FISH detection.

## 5. Conclusions

Our results suggest that TNAC is a special subtype of triple-negative breast cancer with better short-term prognosis despite poor response to neoadjuvant chemotherapy. Further research is needed to assess the possible role of chemotherapy de-escalation and the adding of anti-androgen receptor therapy in the management of TNAC.

## Figures and Tables

**Table 1:** Clinicopathological features of TNAC and TNBC-NA patients who did not receive neoadjuvant therapy.

	TNAC	TNBC-NA	*p*
Age at diagnosis (y)			1.000
20–49	6 (30.0)	6 (30.0)	
50–69	11 (55.0)	11 (55.0)	
70–89	3 (15.0)	3 (15.0)	
T stage			0.196
T1	16 (80.0)	11 (55.0)	
T2	4 (20.0)	8 (40.0)	
T3	0 (0)	0 (0)	
T4	0 (0)	1 (5.0)	
N stage			
N0	18 (90.0)	14 (70.0)	0.348
N1	1 (5.0)	4 (20.0)	
N2		1 (5.0)	
N3	1 (5.0)	1 (5.0)	
AJCC stage			0.323
IA	14 (70.0)	10 (50.0)	
IIA	5 (25.0)	5 (25.0)	
IIB	0 (0)	3 (15.0)	
IIIA	0 (0)	1 (5.0)	
IIIB	0 (0)	0 (0)	
IIIC	1 (5.0)	1 (5.0)	
Radiation			0.500
No	15 (75.0)	12 (60.0)	
Yes	5 (25.0)	8 (40.0)	
Surgery type			1.000
BCS *	7 (35.0)	6 (30.0)	
Mastectomy	13 (65.0)	14 (70.0)	
Laterality			0.747
Left	7 (35.0)	9 (45.0)	
Right	13 (65.0)	11 (55.0)	
Ki-67 (%)			<0.001
0–29	18 (90.0)	2 (10.0)	
30–59	2 (10.0)	3 (15.0)	
60–99	0 (0.0)	15 (75.0)	
Histologic grade			0.006
I	8 (40.0)	1 (5.0)	
II	5 (25.0)	4 (20.0)	
III	5 (25.0)	15 (75.0)	
Missing	2 (10.0)		
TILs			0.051
0–10	11 (61.1)	6 (30.0)	
11–40	6 (33.3)	7 (35.0)	
41–90	1 (5.6)	7 (35.0)	

* BCS: breast-conserving surgery.

**Table 2:** Clinicopathological features of TNAC and TNBC-NA patients who received neoadjuvant therapy.

	TNAC	TNBC-NA	*p*
Age at diagnosis (y)			
20–49	6 (28.6)	6 (28.6)	1.000
50–69	15 (71.4)	15 (71.4)	
70–89	0 (0.0)	0 (0.0)	
T stage			
T1	16 (76.2)	13 (61.9)	0.767
T2	3 (14.3)	5 (23.8)	
T3	1 (4.8)	1 (4.8)	
T4	1 (4.8)	2 (9.5)	
N stage			
N0	16 (76.2)	12 (57.1)	0.065
N1	0 (0)	6 (28.6)	
N2	4 (19.0)	2 (9.5)	
N3	1 (4.8)	1 (4.8)	
AJCC stage			
IA	13 (61.9)	8 (38.1)	0.541
IIA	2 (9.5)	6 (28.6)	
IIB	1 (4.8)	2 (9.5)	
IIIA	3 (14.3)	2 (9.5)	
IIIB	1 (4.8)	2 (9.5)	
IIIC	1 (4.8)	1 (4.8)	
Radiation			
No	12 (57.1)	10 (47.6)	0.757
Yes	9 (42.9)	11 (52.4)	
Surgery type			
BCS	8 (38.1)	6 (28.6)	0.743
Mastectomy	13 (61.9)	15 (71.4)	
Laterality			
Left	14 (66.7)	13 (61.9)	1.000
Right	7 (33.3)	8 (38.1)	
Ki-67 (%)			
0–29	19 (90.5)	4 (19.0)	<0.001
30–59	2 (9.5)	7 (33.3)	
60–99	0 (0.0)	10 (47.6)	
Histologic grade			
I	6 (28.6)	2 (9.5)	0.164
II	11 (52.4)	11 (52.4)	
III	2 (9.5)	7 (33.3)	
Missing	2 (9.5)	1 (4.8)	
TILs			
0–10	14 (73.7)	9 (45.0)	0.097
11–40	2 (10.5)	8 (40.0)	
41–90	3 (15.8)	3 (15.0)	

**Table 3:** Clinical information of TNAC patients who received neoadjuvant therapy.

	Neoadjuvant Therapy	Clinical Evaluation	T Stage	N Stage	MP Grade	RCB	Ki-67 (%)	Histologic Grade	TILs (%)
TNAC-1	ddEC/T1w	Unk/SD	1	0	3	II	5	I	2
TNAC-2	AC-T	Unk	4	2	2	III	30	II	15
TNAC-3	TP1w/CEF/DF	SD/SD/PR	1	0	2	II	20	I	5
TNAC-4	TP/CEF/NP	SD/SD/PR	1	2	2	III	10	II	40
TNAC-5	TPX	PR	1	0	2	II	5	II	8
TNAC-6	TP1w	PR	1	0	4	I	10	II	1
TNAC-7	ddEC/T1w	SD/PR	1	0	3	II	20	II	1
TNAC-8	TP1w	SD	1	0	3	II	10	II	60
TNAC-9	CEF/TP1w	Unk/PR	1	0	3	I	10	I	3
TNAC-10	T1w/EC	Unk/PR	2	0	4	II	5	II	5
TNAC-11	T1w/AC	Unk/SD	2	2	2	III	15	II	10
TNAC-12	ddEC/ddT175	Unk	1	0	2	II	20	I	5
TNAC-13	TPX/AC	Unk/PR	1	0	3	II	15	I	0
TNAC-14	CEF	SD	1	2	2	III	20	Unk	Unk
TNAC-15	EC	SD	1	0	2	II	20	II	3
TNAC-16	TP/NE/DCF/NP	Unk/Unk/Unk/PR	1	0	2	II	5	I	0
TNAC-17	CEF/TP1w	Unk/PR	1	3	2	III	25	III	3
TNAC-18	ddEC/ddT175	Unk/PR	1	0	3	II	20	II	45
TNAC-19	ddEC/T1w	Unk/SD	2	0	3	II	5	II	80
TNAC-20	ddEC/T1w	PR/SD	1	0	3	II	40	Unk	Unk
TNAC-21	TX	SD	3	0	1	II	15	III	2

Abbreviations: A—doxorubicin; C—cyclophosphamide; D—daunorubicin; E—epirubicin; F—fluorouracil; N—vinorelbine; P—platin; T—docetaxel or paclitaxel; Unk—unknown; X—Xeloda.

**Table 4:** Baseline information of TNAC patients from the SEER database.

	Before PSM			After PSM		
	No Chemo	Chemo	*p*-Value	No Chemo	Chemo	*p*-Value
Sample size	151	291		87	87	
Age group			<0.001			1.000
<50 years	5 (3.3)	40 (13.7)		3 (3.4)	3 (3.4)	
50–69 years	41 (27.2)	191 (65.6)		36 (41.4)	36 (41.4)	
70+ years	105 (69.5)	60 (20.6)		48 (55.2)	48 (55.2)	
Income			0.579			0.456
USD 50,000–69,999	74 (49.0)	129 (44.3)		43 (49.4)	35 (40.2)	
USD 70,000+	56 (37.1)	113 (38.8)		30 (34.5)	34 (39.1)	
<USD 50,000	21 (13.9)	49 (16.8)		14 (16.1)	18 (20.7)	
Race			0.542			0.765
Hispanic (All races)	11 (7.3)	25 (8.6)		7 (8.0)	6 (6.9)	
Non-Hispanic Asian or Pacific Islander	19 (12.6)	37 (12.7)		13 (14.9)	16 (18.4)	
Non-Hispanic Black	26 (17.2)	40 (13.7)		13 (14.9)	9 (10.3)	
Non-Hispanic White	95 (62.9)	185 (63.6)		54 (62.1)	56 (64.4)	
Others	0 (0.0)	4 (1.4)		0 (0.0)	0 (0.0)	
Stage			<0.001			1.000
I	81 (53.6)	68 (23.4)		44 (50.6)	44 (50.6)	
II	25 (16.6)	92 (31.6)		18 (20.7)	18 (20.7)	
III	5 (3.3)	27 (9.3)		3 (3.4)	3 (3.4)	
IV	1 (0.7)	5 (1.7)		0 (0.0)	0 (0.0)	
Unk	39 (25.8)	99 (34.0)		22 (25.3)	22 (25.3)	
Grade			<0.001			1.000
Well-differentiated; Grade I	21 (13.9)	12 (4.1)		8 (9.2)	8 (9.2)	
Moderately differentiated; Grade II	90 (59.6)	159 (54.6)		62 (71.3)	62 (71.3)	
Poorly differentiated; Grade III	34 (22.5)	106 (36.4)		16 (18.4)	16 (18.4)	
Undifferentiated; anaplastic; Grade IV	1 (0.7)	0 (0.0)		0 (0.0)	0 (0.0)	
Unk	5 (3.3)	14 (4.8)		1 (1.1)	1 (1.1)	
Sequence			0.926			0.316
1st of 2 or more primaries	19 (12.6)	39 (13.4)		12 (13.8)	18 (20.7)	
One primary only	132 (87.4)	252 (86.6)		75 (86.2)	69 (79.3)	
Surgery			0.033			1.000
No	9 (6.0)	5 (1.7)		0 (0.0)	0 (0.0)	
Yes	142 (94.0)	286 (98.3)		87	87	
Radiation			0.019			1.000
No	77 (51.0)	113 (38.8)		39 (44.8)	39 (44.8)	
Yes	74 (49.0)	178 (61.2)		48 (55.2)	48 (55.2)	

## Data Availability

All data generated or analyzed during this study are included in this published article.

## Supplementary Materials

The following supporting information can be downloaded at: https://www.mdpi.com/article/10.3390/jcm11061607/s1, Table S1: Clinical information of TNBC-NA patients who received neoadjuvant therapy.
