# Supplementary material for: Triple-Negative Apocrine Breast Carcinoma Has Better Prognosis despite Poor Response to Neoadjuvant Chemotherapy
Source: J Clin Med. 2022 Mar 14;11(6):1607. doi: 10.3390/jcm11061607 (PMC8949126; doi:10.3390/jcm11061607)
Supplement: Supplementary file 1 [file jcm-11-01607-s001.zip › jcm-1582920-supplementary.pdf]

Supplementary Table S1. Clinical information of TNBC-NA patients who received neoadjuvant therapy.

	Neoadjuvant therapy	Clinical evaluation	T stage	N stage	MP grade	Ki67(%)	Histologic grade	TILs(%)	RCB index
TNBC-1	ddEC	PR	2	0	5	30	II	45	0
TNBC-2	TP1w/CEF	PR/PR	2	0	2	5	I	2	II
TNBC-3	TP/CEF	PR	1	0	3	75	II	20	II
TNBC-4	ddEC/T1w	PR	1	3	2	15	I	20	III
TNBC-5	CEF	PD	3	1	3	50	III	1	III
TNBC-6	TP	PR	1	2	3	90	II	25	II
TNBC-7	ddEC/ddT175	PR/Unk	1	1	3	40	II	35	II
TNBC-8	TP1w/CEF	Unk	1	0	3	10	III	90	II
TNBC-9	TP1w/TX/AC/NB	SD/SD/PD/PR	4	0	2	60	III	25	II
TNBC-10	T1w	PR	1	0	5	60	III	5	0
TNBC-11	T1w	SD	1	0	3	5	II	5	II
TNBC-12	ddEC/ddT	SD/SD	1	1	3	30	II	15	II
TNBC-13	ddEC/ddT175	SD/Unk	2	1	2	75	II	20	III
TNBC-14	CEF	PR	1	0	2	75	III	35	II
TNBC-15	TF/NP	PD/PR	2	0	2	60	III	0	II

TNBC-16	TP/CEF/NP	PR/PR/CR	1	0	3	40	II	70	II
TNBC-17	T1w/CEF/NP	PR	1	0	3	40	II	2	II
TNBC-18	EC/TX	PR/PR	1	1	3	30	II	2	II
TNBC-19	TX/NP	SD/PR	4	2	4	80	II	10	II
TNBC-20	ddEC/T1w	PR/PR	1	0	5	70	III	5	0
TNBC-21	TX/EC	SD/PR	2	1	5	75	Unk	Unk	0

Abbreviations: A—doxorubicin; B—bevacizumab; C—cyclophosphamide; D—daunorubicin; E—epirubicin; F— fluorouracil; N—vinorelbine; P—platin; T—docetaxel or paclitaxel; Unk-Unknown; X—xeloda.
